# Telehealth Versus Face-to-face Psychotherapy for Less Common Mental Health Conditions: Systematic Review and Meta-analysis of Randomized Controlled Trials

**DOI:** 10.2196/31780

**Published:** 2022-03-11

**Authors:** Hannah Greenwood, Natalia Krzyzaniak, Ruwani Peiris, Justin Clark, Anna Mae Scott, Magnolia Cardona, Rebecca Griffith, Paul Glasziou

**Affiliations:** 1 Institute for Evidence-Based Healthcare Bond University Robina Australia; 2 School of Pharmacy University of Queensland Brisbane Australia; 3 Gold Coast University Hospital Evidence-Based Practice Professorial Unit Southport Australia; 4 Private Practice Clinical Psychology Gold Coast Australia

**Keywords:** telemedicine, psychology, mental health, psychotherapy, primary health care, behavioral sciences, systematic review

## Abstract

**Background:**

Mental disorders are a leading cause of distress and disability worldwide. To meet patient demand, there is a need for increased access to high-quality, evidence-based mental health care. Telehealth has become well established in the treatment of illnesses, including mental health conditions.

**Objective:**

This study aims to conduct a robust evidence synthesis to assess whether there is evidence of differences between telehealth and face-to-face care for the management of less common mental and physical health conditions requiring psychotherapy.

**Methods:**

In this systematic review, we included randomized controlled trials comparing telehealth (telephone, video, or both) versus the face-to-face delivery of psychotherapy for less common mental health conditions and physical health conditions requiring psychotherapy. The psychotherapy delivered had to be comparable between the telehealth and face-to-face groups, and it had to be delivered by general practitioners, primary care nurses, or allied health staff (such as psychologists and counselors). Patient (symptom severity, overall improvement in psychological symptoms, and function), process (working alliance and client satisfaction), and financial (cost) outcomes were included.

**Results:**

A total of 12 randomized controlled trials were included, with 931 patients in aggregate; therapies included cognitive behavioral and family therapies delivered in populations encompassing addiction disorders, eating disorders, childhood mental health problems, and chronic conditions. Telehealth was delivered by video in 7 trials, by telephone in 3 trials, and by both in 1 trial, and the delivery mode was unclear in 1 trial. The risk of bias for the 12 trials was low or unclear for most domains, except for the lack of the blinding of participants, owing to the nature of the comparison. There were no significant differences in symptom severity between telehealth and face-to-face therapy immediately after treatment (standardized mean difference [SMD] 0.05, 95% CI −0.17 to 0.27) or at any other follow-up time point. Similarly, there were no significant differences immediately after treatment between telehealth and face-to-face care delivery on any of the other outcomes meta-analyzed, including overall improvement (SMD 0.00, 95% CI −0.40 to 0.39), function (SMD 0.13, 95% CI −0.16 to 0.42), working alliance client (SMD 0.11, 95% CI −0.34 to 0.57), working alliance therapist (SMD −0.16, 95% CI −0.91 to 0.59), and client satisfaction (SMD 0.12, 95% CI −0.30 to 0.53), or at any other time point (3, 6, and 12 months).

**Conclusions:**

With regard to effectively treating less common mental health conditions and physical conditions requiring psychological support, there is insufficient evidence of a difference between psychotherapy delivered via telehealth and the same therapy delivered face-to-face. However, there was no includable evidence in this review for some serious mental health conditions, such as schizophrenia and bipolar disorders, and further high-quality research is needed to determine whether telehealth is a viable, equivalent treatment option for these conditions.

## Introduction

### Background

Worldwide, mental health disorders are a leading cause of distress and disability, with 1 in every 4 people expected to be personally impacted throughout their lifetime [1]. Some evidence suggests that mental health difficulties may be increasing; a previous systematic review found a small but significant increase in mental illness prevalence rates from 1978 to 2015, although the authors note that this may have been driven by demographic changes across this period [2]. In addition, the emergence of COVID-19 has seen mental health adversely impacted worldwide [3,4]. This seems to indicate that this already debilitating problem may become a further global burden in the future. Thus, it seems crucial for quality mental health support to be widely available to the public in a safe and accessible way.

Although telehealth was available and suggested to be effective for psychotherapy before the COVID-19 pandemic [5], its uptake was somewhat limited within the delivery of psychological services [6]. A study in the United States found that before the COVID-19 pandemic, psychologists were hesitant to use telehealth owing to lack of training, concerns for client safety, and privacy, among other concerns [7]. In addition, a qualitative study of mental health professionals highlighted concerns around the quality of the patient-therapist relationship [8]. Given the health risks posed by face-to-face meetings, especially for older people or otherwise vulnerable, there was a rapid shift to remote delivery in health care services worldwide [9-12].

Although the pandemic was the catalyst that thrust telehealth to the forefront of health care delivery, there are many advantages to telehealth service provision for mental health. Telehealth extends care to patients with limited access to in-person therapy, including those in rural and remote areas. A narrative review examining telehealth access in rural communities in the United States found telehealth to be a convenient and efficient way to treat patients, and participants reported acceptability and satisfaction with telehealth services [13]. Furthermore, telehealth also offers a safe and effective option for those who may have access issues or face stigmatization [14]. For some conditions, such as substance use disorder, access to therapy delivered remotely may increase engagement with treatment services among groups who would not otherwise attend therapy [15]. For patients being treated for substance abuse, video-delivered treatment was preferred to face-to-face treatment, mostly because of convenience and increased confidentiality [16]. Taken together, the availability of telehealth facilitates increased access of care to those unable or unwilling to engage in face-to-face therapy and promotes continued therapeutic engagement owing to its flexibility and privacy.

Telehealth may also enhance care accessibility for those requiring specialized therapies or those with less common mental health conditions that may not be treated by all clinicians. The skills needed to effectively treat those with less common or more complex mental health conditions or to adequately deliver less common therapy types may require additional training, guided supervision, professional development, or years of clinical experience. This is further compounded in rural and remote areas, where health care disparity is well documented [17-19]. Telehealth presents a potentially effective medium to connect patients requiring specialized forms of care with relevant, qualified therapists.

### Objectives

Evidence supports the use of telehealth for application in some psychotherapies [5,20,21] and the management of common mental health conditions, including reviews in this series for depression (Scott AM et al, PhD, unpublished data, February 2022), anxiety [22], and posttraumatic stress disorder (PTSD) [23]. It is important to rigorously assess whether its effectiveness is generalizable beyond these groups. The aim of this systematic review is to assess whether there are any differences between telehealth-based psychotherapy and face-to-face psychotherapy across outcomes (patient, process, and cost) for less common mental health conditions (eg, substance use disorder, eating disorders, or childhood disorders) and physical conditions requiring psychological support (eg, cancer or chronic fatigue syndrome).

## Methods

### Overview

We aim to find, appraise, and synthesize studies that compared psychotherapy delivered via telehealth (video, telephone, or both) versus face-to-face for patients of any age in the primary health care setting. This systematic review is reported following the PRISMA (Preferred Reporting Items for Systematic Reviews and Meta-Analyses) 2009 statement [24], and the review protocol was developed prospectively.

### Inclusion and Exclusion Criteria

#### Study Design

We included randomized controlled trials (RCTs) of any design (eg, parallel, cluster, crossover, factorial, or mixed), which included >10 patients. We excluded all other study designs, such as controlled nonrandomized trials, qualitative studies, and observational studies (cohort, case-control, cross-sectional, case series, and case reports).

#### Participants

We included studies with people of any age or gender, who were receiving psychotherapy for less common mental health conditions, such as bulimia nervosa and substance use disorder, or any conditions where psychotherapy was used, such as cognitive behavioral therapy (CBT) for patients with cancer with high psychological needs. Although anxiety [22], depression (Scott AM et al, PhD, unpublished data, February 2022), and PTSD [23] were within the scope of this review, there was enough literature to conduct separate systematic reviews by condition, and hence, these were excluded. Studies involving hospital patients (eg, explicitly identified as taking place in hospital wards, or with patients shortly after discharge) or those consulting a secondary or tertiary specialist (ie, a psychiatrist) were excluded. Studies in hospital-discharged patient populations that explicitly identified the provision of therapy by a psychologist, therapist, psychotherapist, or counselor, however, were included.

#### Interventions

We included studies of interventions involving standard care psychological therapies for mental health conditions or physical conditions where psychological therapy was required, including but not limited to CBT, parent-child interaction therapy, cognitive behavioral intervention for tics, and parent training. Studies examining novel treatments for mental health were excluded.

#### Comparators

We included studies with an equivalent face-to-face comparator or other telehealth comparators (ie, video intervention with a telephone comparator). The intervention and comparator had to deliver a similar or identical level of care (ie, care similar in intensity, frequency, and duration). Studies with a comparator that included a wait-list control or clinically inequivalent active comparator were excluded.

#### Outcomes (Primary and Secondary)

The primary patient outcome was global or symptom severity. The secondary patient outcomes included improvement in psychological symptoms and functioning. The tertiary process (working alliance and satisfaction) or financial (cost) outcomes are included but reported in Multimedia Appendix 1. Studies that met other inclusion criteria but did not report on one of the primary or secondary outcomes were included and reviewed. This is important to distinguish, as we either meta-analyzed outcomes or summarized them narratively if meta-analysis was not possible.

### Search Strategy to Identify Studies

#### Database Search for Primary Studies

The following databases were searched from inception until November 18, 2020: PubMed (MEDLINE), Embase, and CENTRAL via the Cochrane Library. The original search string (Multimedia Appendix 2) was designed in PubMed and translated for use in other databases using the Institute for Evidence-Based Healthcare’s Polyglot Search Translator, an automation tool designed to translate search strings between databases [25]. This included a number of concepts and variants, such as *Telemedicine* AND *Primary healthcare* AND *face-to-face* AND *randomised*. On January 11, 2021, we conducted a backward (cited) and forward (citing) citation analysis using the web-based citation database Scopus [26] on included studies identified during previous searches. These were screened against the inclusion criteria.

#### Restriction on Publication Type

No restrictions by language or publication date were imposed. We included only those publications from RCTs that were published in full. We excluded publications available as abstract only (eg, conference abstract) or with no additional results information available (eg, from a clinical trial registry record).

### Study Selection and Screening

**Figure 1:**
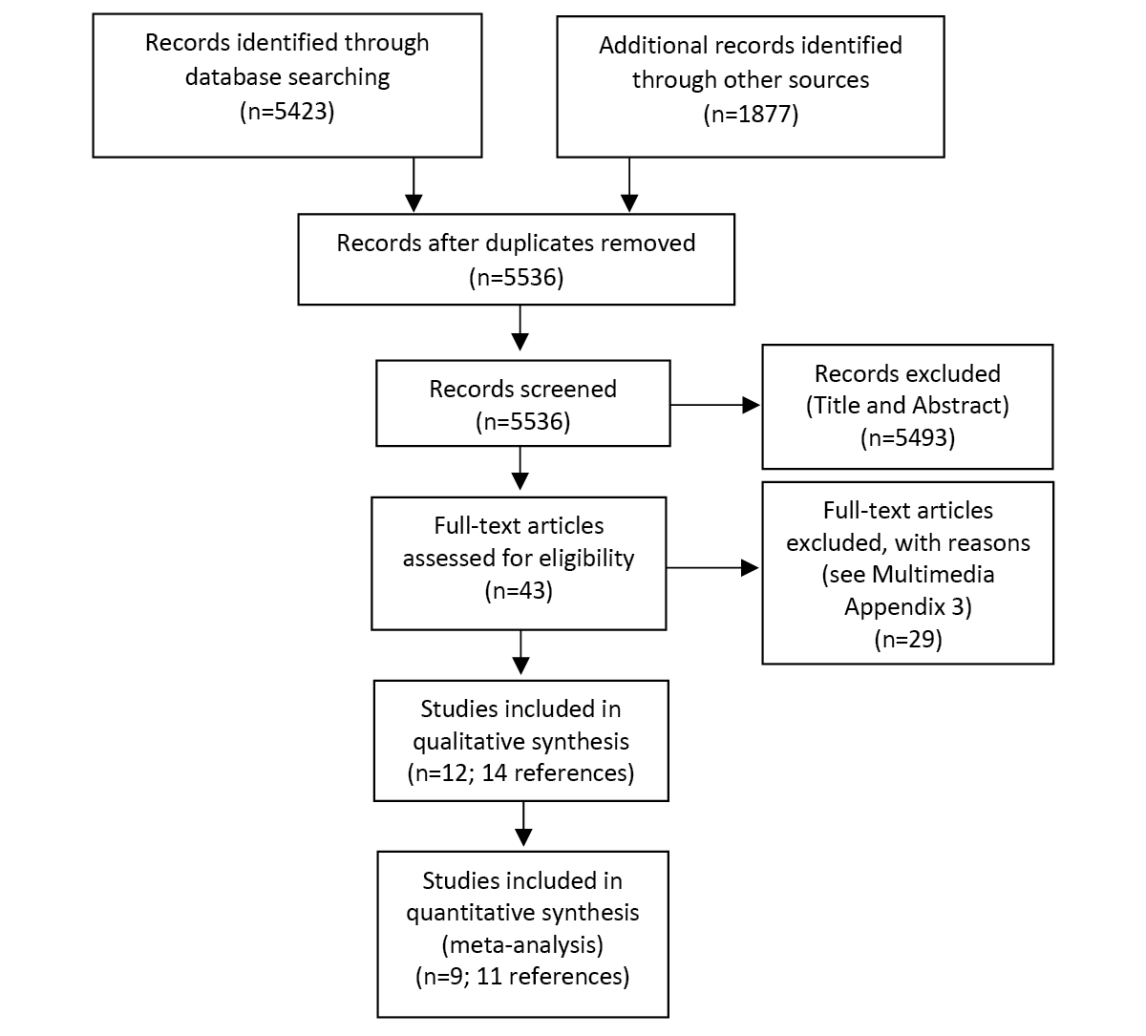
PRISMA (Preferred Reporting Items for Systematic Reviews and Meta-Analyses) flowchart.

Titles and abstracts were screened independently by author pairs (AMS, RP, MC, JC, NK, HG, and PG) against the inclusion criteria. In addition, 1 author (JC) retrieved full texts, and 2 authors (HG and NK) screened the full texts for inclusion. Any disagreements were resolved by discussion or reference to the third screener. The forward backward citation analysis was conducted by 1 author (JC) and screened by 3 authors (HG, NK, and RP), and full text was obtained by HG. The study selection process for includable studies is reported in the PRISMA flow diagram (Figure 1), and studies excluded at the full-text screening stage are in Multimedia Appendix 3 with reasons for exclusion.

### Data Extraction

We used 2 data extraction forms for study characteristics and outcome data, which were piloted on 2 studies in the review. Data from the included studies were extracted independently by 2 authors (NK and HG) into the data extraction forms (Textbox 1), and discrepancies were resolved by discussion or by reference to a third author.

**List of extracted information.**

**Extracted information**

- Methods: Study authors, country, design, and duration of follow-up—as reported
- Participants: n, condition needing psychotherapy, randomization, age (years), mean (SD)
- Interventions: telehealth—provider, therapy, and dose
- Comparators: face-to-face—provider, therapy, and dose
- Outcomes: n, mean (SD), and *P* value (or as reported by authors)—patient (global or symptom severity, improvement in psychological symptoms, and functioning), process (working alliance and satisfaction), and financial (cost)

### Risk of Bias in Included Studies

A total of 2 review authors (HG and NK) independently assessed the risk of bias for the included studies using the Cochrane Collaboration Risk of Bias Tool 1, as outlined in the Cochrane Handbook [27], and all disagreements were resolved by discussion.

The following domains were assessed for possible bias: (1) random sequence generation, (2) allocation concealment, (3) the blinding of participants and personnel, (4) the blinding of the outcome assessment, (5) incomplete outcome data, (6) selective outcome reporting, and (7) other bias (focusing on potential biases due to funding or conflict of interest).

Each domain was graded as low, high, or unclear, including quote or summary from the relevant trial, which summarized why the grading was applied.

### Data Synthesis and Analysis

Review Manager 5.4, the Cochrane Collaboration tool for conducting meta-analyses and creating forest plots, was used to calculate the treatment effect [28]. As all outcome measures were continuous, we used mean difference or standardized mean difference (SMD). We performed meta-analyses only when possible (when ≥2 studies or comparisons reported the same or similar outcome) and where appropriate data were available that allowed us to calculate the SMD. Where these data were not available and thus meta-analysis was not possible, we narratively report the results. We anticipated a considerable heterogeneity between studies and used a random-effects model.

The unit of analysis was the individual, which was available for every study in this review. We did not contact study authors to provide missing data. We used the *I*^2^ statistic to examine the heterogeneity of the included studies. Subgroup analyses were conducted according to the duration of follow-up: posttreatment and 3, 6, and 12 months.

As <10 trials were included in any data synthesis, we did not create a funnel plot, and sensitivity analyses were not conducted. We planned to conduct a subgroup analysis of gender, setting, age, and sensitivity by including or excluding studies at high risk of bias; however, the low number of included studies did not allow for this.

## Results

### Search Results

The primary study search found 5423 references, and 1877 additional references were found in the forward and backward citation search and clinical trial registries. After deduplication, 5536 records were screened in title and abstract. A total of 5493 references were excluded, and 43 full texts were assessed for inclusion. Moreover, 12 RCTs (across 14 articles) were included in this systematic review, and 9 were able to be meta-analyzed (Figure 1). We found 2 potentially relevant but still in-progress clinical trials (Multimedia Appendix 4 [29,30]).

### Characteristics of Included Studies

Of the 12 included RCTs, 10 (83%) were conducted in the United States, and the other 2 (17%) studies were conducted in the United Kingdom. A total of 931 patients were included in aggregate. Studies have examined psychotherapy delivered for a variety of less common mental health conditions and other conditions requiring psychotherapy. Of the 12 studies, 2 (17%) included patients with type 1 diabetes mellitus, 2 (17%) included patients with addiction disorders, 1 (8%) (reported in 4 articles) treated patients with bulimia nervosa or eating disorder not otherwise specified, 3 (25%) studies included participants with children’s disorders (including disruptive behavior disorder, tic disorders, and attention-deficit hyperactivity disorder), 2 (17%) included patients with chronic illness (chronic fatigue syndrome and chronic multisymptom illness), 1 (8%) study included patients with a range of mental health conditions, and 1 (8%) included patients with cancer who had high psychological needs. The types of therapies varied by target condition: of the 12 studies, 5 (42%) used CBT, 4 (33%) used a family therapy (parent-child interaction therapy, parent training, and behavioral family systems therapy for diabetes), 2 (17%) used addiction therapies (opioid treatment program and acute therapy service), and 1 (8%) used a cognitive behavioral intervention for tics. Finally, of the 12 studies, 3 (25%) used the telephone to deliver telehealth, 7 (58%) used video, and 1 (8%) had included video and telephone groups, and in 1 (8%) study, it was unclear whether video or telephone was used. All studies compared the telehealth intervention to face-to-face intervention (Table 1).

**Table 1:** Characteristics of included studies.

Reference	Country	RCT^a^ design	Follow-up (months)	Study participants, total N (n TH^b^, n F2F^c^)	Participants	Age (years), mean (SD)	Intervention	Telehealth: modality dose	Comparator: modality dose
Burgess et al [31]	United Kingdom	Parallel, 2 arm	12	80 (45, 35)	Adults (aged 18-65 years) with chronic fatigue syndrome (comorbidities excluded)	37.4 (10.1).	CBT^d^	Telephone, 3-hour 1 × F2F; 30 minutes, 13 sessions, fortnightly	F2F, 3-hour 1 x F2F; 50-60 minutes, 13 sessions
Comer et al [32]	United States	Parallel, 2 arm	6	40 (20, 20)	Children (aged 3-5 years) with principal diagnosis disruptive behavior disorder (serious comorbidities excluded) and their parents or caregivers	4.0 (0.9)	Parent-child interaction therapy	Video, until mastery was achieved, mean sessions 21.7	F2F until mastery was achieved, mean sessions 20.8
Day and Schneider [33]	United States	Parallel, 3 arm	None	91 (completers only reported—26 video, 27 telephone, and 27 F2F)	Adults (aged 19-75 years) presenting with any mental health issue to a community counseling center	39.3 (15.9)	CBT	Video and 2-way audio (telephone analogous), 5 sessions	F2F, 5 sessions
Duke et al [34]	United States	Parallel, 2 arm	3	90 (46, 44)	Adolescents (aged 12-19 years) with type 1 diabetes (uncontrolled comorbidities excluded) and their caregivers	15.0 (1.75)	Behavioral family systems therapy for diabetes	Video, 60-90 minutes, up to 10× sessions, 12 weeks	F2F, 60-90 minutes, up to 10× sessions, 12 weeks
Freeman et al [35]	United States	Parallel, 2 arm	None	92 (47, 45)	Adolescents (aged 12-19 years) with poorly controlled type 1 diabetes (no comorbidity exclusion) and 1 parent or legal guardian	TH 14.9 (1.9); F2F 15.2 (1.8)	Behavioral family systems therapy for diabetes	Video, 60-90 minutes, up to 10× sessions, 12 weeks	F2F, 60-90 minutes, up to 10× sessions, 12 weeks
Himle et al [36]	United States	Parallel, 2 arm	4	20 (10, 10)	Children (aged 8-17 years) who met DSM^e^ criteria for Tourette or chronic tic disorder with or without comorbidities	TH 11.3 (2.3); F2F 12 (3.3)	Cognitive behavioral intervention for tics	Video, 6× weekly sessions+2× biweekly sessions, 10 weeks	F2F, 6× weekly session+2× biweekly sessions, 10 weeks
King et al [37]	United States	Parallel, 2 arm	3	85 (50, 35)	Adult outpatients receiving opioid dependence treatment (no comorbidity exclusion)	TH 40.5 (11.2); F2F 41.1 (10.5)	Opioid treatment program	Video, 30-40 minutes, 12× weekly sessions, 12 weeks	F2F, 30-40 minutes, 12× weekly session, 12 weeks
King et al [16]	United States	Parallel, 2 arm	None	37 (20, 17)	Adult outpatients with a partial response to methadone maintenance treatment (no comorbidity exclusion)	TH 42.7; F2F 41.4	Acute therapy service	Video, 1 hour, 2× sessions, 6 weeks	F2F, 1 hour, 2× sessions, 6 weeks
McAndrew et al [38]	United States	Parallel, 3 arm	12	128 (42, 43; 43 UC^f^)	Adult veterans with chronic multisymptom illness (serious psychiatric and medical comorbidities excluded)	TH 57.6 (6.6); F2F 55.4 (8.2)	CBT	Telephone, up to 10 sessions	F2F, up to 10 sessions
Crow et al [39], Ertelt et al [40], Mitchell et al [41]	United States	Parallel, 2 arm	12	128 (62, 66)	Adults (aged >18 years) with bulimia nervosa (including comorbidities but excluding suicidal ideation, psychosis, schizophrenia and bipolar)	TH 28.4 (10.4); F2F 29.6 (10.9)	CBT	Unclear, 20 sessions, 16 weeks	F2F, 20 sessions, 16 weeks
Watson et al [42]	United Kingdom	Parallel, 2 arm	None	118 (60, 58)	Adults (aged 18-79 years) with a cancer diagnosis and comorbid high psychological needs	TH 48.5 (13.3); F2F 52.4 (13.1)	CBT	Telephone, 8 sessions, 12 weeks	F2F, 8 sessions, 12 weeks
Xie et al [43]	United States	Parallel, 2 arm	None	22 (9, 13)	Children (aged 6-14) with primary diagnosis ADHD^g^ (excluding unstable medical conditions and other serious psychiatric disorders) and their parents	10.4 (NR^h^)	Parent training	Video, 10 weekly session, 10 weeks	F2F, 10 weekly sessions, 10 weeks

^a^RCT: randomized controlled trial.

^b^TH: telehealth.

^c^F2F: face-to-face.

^d^CBT: cognitive behavioral therapy.

^e^DSM: Diagnostic and Statistical Manual of Mental Disorders.

^f^UC: usual care.

^g^ADHD: attention-deficit/hyperactivity disorder.

^h^NR: not reported.

### Risk of Bias

**Figure 2:**
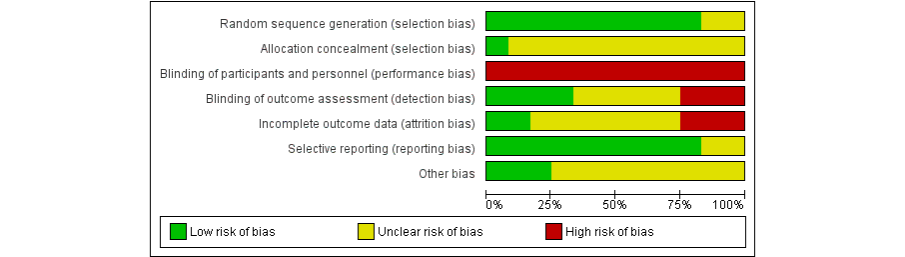
Risk of bias graph: review authors’ judgments about each risk of bias item presented as percentages across all included studies.

Overall, of the 12 studies, 10 (83%) adequately reported on random sequence generation and selective reporting. Declarations of conflicts of interest and funding (reported under other bias) were adequately reported for only 25% (3/12) of the studies, with the remaining 75% (9/12) not reporting this clearly. Allocation concealment was not clearly reported in most studies, with only 8% (1/12) of the studies reporting this satisfactorily. The blinding of the outcome assessment and incomplete outcome data were at high risk of bias for 25% (3/12) of the studies, with the remaining 75% (9/12) of the studies rated at either unclear or low risk of bias. Notably, the blinding of participants and personnel was a high bias risk for all 12/12 (100%) studies, as the telehealth versus face-to-face nature of the interventions was incompatible with blinding (Figure 2).

### Primary Outcome: Global or Symptom Severity

A total of 6 scales across 7 studies were used to report outcomes related to symptom severity (see Multimedia Appendix 5 for a summary of scales used).

**Figure 3:**
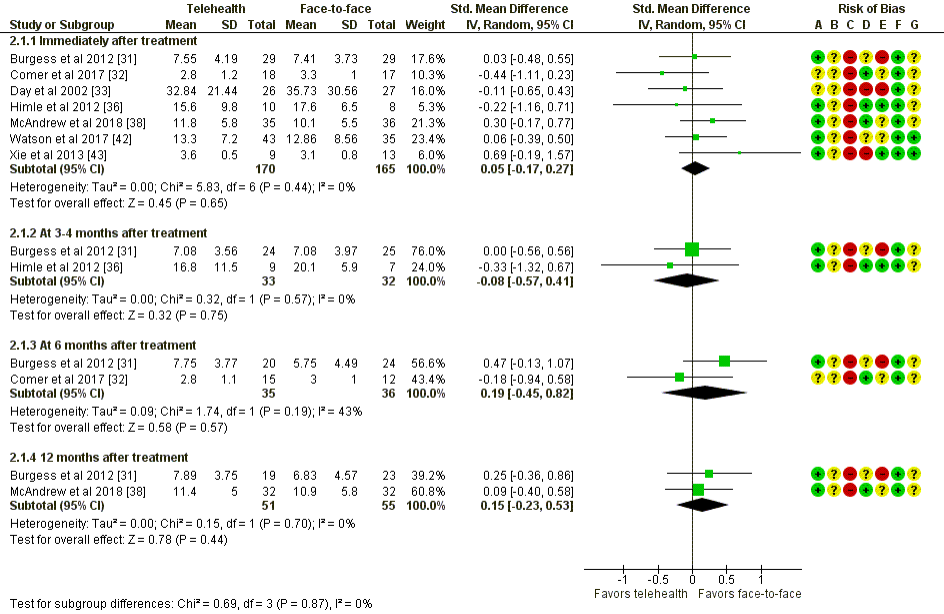
Telehealth versus face-to-face for mental conditions: assessment of symptom severity. Std: standard. [31-33, 36, 38, 42, 43].

In addition, 7 studies reported sufficient data for this outcome and were able to be pooled and meta-analyzed (Figure 3). Data were available for four time point subgroups—immediately after treatment and 3- to 4-, 6-, and 12-month follow-ups.

There were no significant differences in severity outcomes between telehealth and face-to-face therapy immediately after treatment (335 participants; mean difference 0.05, 95% CI −0.17 to 0.27; *P=*.65) or at any of the follow-up time points, including 3 to 4 months (65 participants; SMD −0.08, 95% CI −0.57 to 0.41; *P=*.75), 6 months (71 participants; SMD 0.19, 95% CI −0.45 to 0.82; *P=*.57), and 12 months (106 participants; SMD 0.15, 95% CI −0.23 to 0.53; *P=*.44).

There was moderately high heterogeneity reported for the 6-month follow-up subgroup (*I*^2^=43%).

### Secondary Outcomes

#### Improvement

A total of 3 different scales were used to describe patients’ overall improvement across the studies (see Multimedia Appendix 5 for a summary of scales used).

**Figure 4:**
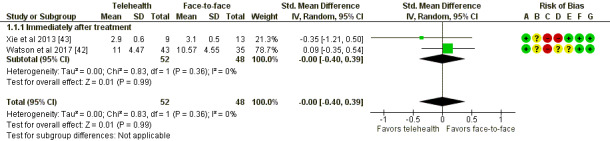
Telehealth versus face-to-face for mental conditions: assessment of improvement of psychological symptoms. Std: standard. [42, 43].

In addition, 2 studies were able to be meta-analyzed; the remaining 3 studies are reported narratively. These 2 meta-analyzed studies involved a total of 100 participants (Figure 4). Data were available at one time point; that is, immediately after treatment. There was no evidence of difference between the 2 groups in this comparison, with an SMD of −0 (95% CI −0.4 to 0.39; *P=*.99).

Burgess et al [31] reported global improvement on a self-rated 6-item scale, ranging from very much better to very much worse. Among 29 telehealth participants immediately after treatment, 14 (48%) rated their improvement as very much or much better, whereas 15 (52%) rated their improvement as a little better to very much worse. For 28 face-to-face participants immediately after treatment, 15 (54%) rated themselves as improved, whereas 13 (46%) rated themselves as only a little better or worse. Although this is variable at the 6- and 12-month follow-up time points, there were no differences between groups at any time point (at 6 months, 8/20, 40% telehealth participants and 15/25, 60% face-to-face participants rated themselves as very much or much better, and at 12 months, 11/20, 55% telehealth and 13/23, 57% face-to-face participants rated themselves as very much or much better).

Comer et al [32] and Himle et al [36] both reported using the Clinical Global Impression-Improvement scale, reporting the percentage of participants who received a score of 1 or 2 (very much improved or much improved). Among participants in the study by Comer et al [32], of the 14 participants in the telehealth group, 12 (86%) had improvement, whereas of the 14 participants in the face-to-face group, 11 (79%) improved. Furthermore, at 6 months after treatment, 83% (10/12) of the telehealth participants and 73% (8/11) of the face-to-face participants still reported very much or much improvement. It is unclear whether the differences between groups were significant, and the outcomes were reported only for treatment completers, not all participants. The findings from Himle et al [36] are similar at immediately after treatment: 80% (8/10) of the telehealth participants were very much or much improved, whereas 75% (6/8) of the face-to-face participants were improved. However, at follow-up, 56% (5/9) of the telehealth participants and 44% (3/7) of the face-to-face participants were very much or much improved.

#### Function

The outcome was assessed using 4 different scales (see Multimedia Appendix 5 for a summary of scales used).

**Figure 5:**
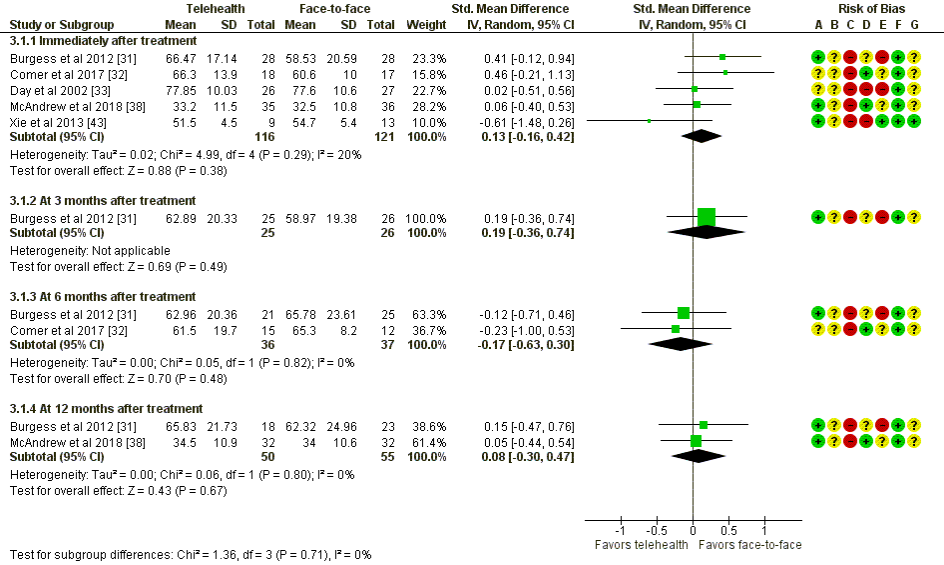
Telehealth versus face-to-face for mental conditions: assessment of functioning. Std: standard. [31-33, 38, 43].

In addition, 6 studies reported sufficient data for this outcome; 5 were able to be meta-analyzed (Figure 5). There were no significant differences in functioning outcomes between telehealth and face-to-face therapy immediately after treatment (237 participants; mean difference 0.13 (95% CI −0.16 to 0.42; *P=*.38) or at any of the follow-up time points, including 3 months (51 participants; SMD 0.19, 95% CI −0.36 to 0.74; *P=*.49), 6 months (73 participants; SMD −0.17, 95% CI −0.63 to 0.3; *P=*.48), and 12 months (105 participants; SMD 0.08, 95% CI −0.3 to 0.47; *P=*.67).

Mitchell et al [41] also reported a function measure using the 36-Item Short-Form Health Survey, reporting on both the mental and physical subscales. At immediately after treatment, there was no difference on the physical subscale between telehealth (41 participants; 54.1, SD 7.9) and face-to-face groups (39 participants; 56.2, SD 5.7). For the mental health subscale, there was no difference between groups for telehealth (41 participants; 42.9, SD 12.6) and face-to-face treatment (39 participants; 45.5, SD 11.9). These results were similar for 3 and 6 months after treatment.

### Tertiary Outcomes

#### Process

A total of 5 studies reported client working alliance outcomes (3 meta-analyzed, n=223, immediately after treatment). There was no difference between telehealth and face-to-face therapy; the SMD was 0.11 (95% CI −0.34 to 0.57; *P*=.63). This subgroup had moderate to high levels of heterogeneity (*I*^2^=63%; see Figure S1 in Multimedia Appendix 1). In addition, 2 studies also reported therapist working alliance outcomes (2 meta-analyzed, n=104, immediately after treatment). There was no evidence of difference between telehealth and face-to-face therapy (SMD −0.16, 95% CI −0.91 to 0.59; *P*=.67), and heterogeneity was high (*I*^2^=72%; see Figure S2 in Multimedia Appendix 1).

A total of 7 studies reported client satisfaction outcomes (3 meta-analyzed, n=131, immediately after treatment), and we found no evidence of difference in satisfaction between groups (SMD 0.12, 95% CI −0.3 to 0.53; *P=*.58; see Figure S3 in Multimedia Appendix 1).

More detailed data for process outcomes (working alliance and client satisfaction) are available in Multimedia Appendix 1, including figures and a narrative analysis of included studies that could not be meta-analyzed.

#### Financial

A total of 3 studies reported cost, but no outcomes were able to be meta-analyzed. Please see Multimedia Appendix 1 for a narrative review of financial outcomes.

## Discussion

### Principal Findings

This systematic review of 12 trials shows insufficient evidence of a difference between psychotherapy delivered via telehealth (telephone or video) and face-to-face therapy, when treating less common mental health conditions or physical conditions requiring psychological support. There were no significant differences between telehealth and face-to-face delivery for patient outcomes (symptom severity, symptom improvement, or global function), immediately after treatment, or at any follow-up time point. For process outcomes (working alliance or therapeutic quality and client satisfaction), there was no significant difference between telehealth and face-to-face care delivery for either clients or therapists, although 1 study reported no difference between groups for therapist satisfaction. Although financial outcome data on costs were not meta-analyzable, patients with substance abuse disorder valued telehealth therapy more highly than face-to-face therapy, treatment costs were lower for telehealth than for face-to-face therapy for patients with bulimia nervosa (especially over large geographical areas), and the cost of therapists’ time was equivalent, regardless of delivery mode, for patients with cancer receiving CBT. This suggests that telehealth is at least as cost-effective as face-to-face care and potentially perceived as more valuable by the client. Overall, the risk of bias of included studies was unclear, owing to unclear reporting, and blinding of participants was not possible because of the nature of the interventions.

Although we found no significant differences between telehealth and face-to-face delivery of psychotherapy across any outcome, to assess equivalence between telehealth and face-to-face psychotherapy, CIs around the effect estimate should be examined to determine whether they exclude the minimally important difference [44]. In the absence of a prespecified minimally important difference, we accept Cohen cutoff for a small effect (0.2), whereby a CI between (−0.20 and 0.20) suggests equivalence between telehealth and face-to-face therapy, and a CI outside these bounds indicates that the minimally important difference cannot be excluded and there is the possibility of a small effect favoring one or the other intervention. For the primary outcome, symptom severity (Figure 3), immediately after treatment, the upper-bound CI is >0.2, so it is possible that the true effect favors face-to-face therapy. For 12 months after treatment, the CI ranges from a possible small effect favoring telehealth to a possible medium effect favoring face-to-face therapy. The same could be applied to all other time points and outcomes to assess the evidence for equivalence. Although we can demonstrate that there is insufficient evidence of a difference between telehealth- and face-to-face–delivered psychotherapy, we cannot conclude whether they are equivalent, given that the CIs around the effect size are rarely narrow enough to exclude the minimally important difference. For common mental health conditions, there is evidence that telehealth is an effective modality in the provision of psychological therapy as face-to-face therapy. There is some evidence of equivalence between videoconferencing and face-to-face care for depression [20], anxiety [21,22], PTSD [23], and psychotherapy broadly [5]. Furthermore, there is evidence of telephone-delivered therapy being effective for depression and anxiety [45]. Although these reviews suggest comparability between telehealth and face-to-face psychotherapy delivery, they all included nonrandomized and noncontrolled studies, which may introduce bias. This review shows no evidence of difference in patient, process, or cost outcomes between telehealth and face-to-face psychotherapy across more diverse patient groups.

### Comparison With Prior Work

This review and meta-analysis shows that telehealth psychotherapy may be similar to face-to-face psychotherapy in treating populations with less common mental health disorders or physical conditions that require psychological support. These synthesized findings support previous primary research suggesting that psychotherapy delivered via telehealth for the treatment of mental health conditions may be comparable with conventional face-to-face therapy. A previous review examined the use of video therapy across a range of mental health conditions, including some of the less common conditions reviewed here, and found video-delivered therapy was equivalent to face-to-face care for outcomes of clinical effectiveness, treatment adherence, and patient satisfaction [14]. In line with our narrative findings for financial outcomes, they also found video therapy to be less costly than face-to-face care. This contrasts with a recent scoping review, finding that telehealth service provision across health care in Australia does not routinely reduce the cost of care delivery [46]. Our findings also support the results of a single-arm study conducted in Japan examining video-delivered CBT, which found that this delivery mode is feasible for the treatment of bulimia nervosa and binge-eating disorders. Previous evidence regarding the impact of telehealth on working alliance is mixed. An RCT examining psychologists’ perceptions of therapeutic alliance in videoconferencing found that therapeutic alliance was rated significantly lower for telehealth than for face-to-face care [47]. A survey conducted during the COVID-19 pandemic of psychotherapists’ experience with remote care found that it was “better than expected” but that telehealth care could not be compared with face-to-face care [48]. In contradiction, results from a more recent survey study found that telehealth was widely accepted by primary mental health care providers [49], although this was not specific to delivery of psychotherapy via telehealth. A recent study examining working alliance via telehealth for anxiety disorders found that these clients had a stronger working alliance with their clinician when treated via telehealth [50]. Our findings, using only data from RCTs, support previous research suggesting that working alliance is as strong in telehealth as it is in face-to-face care. However, further research is needed to fully understand how telehealth changes the client and clinician relationship dynamic and how this may change circumstantially based on clinician and client perceptions of telehealth and the patient’s specific treatment needs.

### Strengths and Limitations

This review has many strengths, which add weight to our findings and conclusions. We applied rigorous methodology to find includable studies by establishing a prospective protocol and following PRISMA guidelines. Clear, strict inclusion and exclusion criteria allowed for studies in a variety of different health conditions to be synthesized and systematically reviewed. Further, we only included RCTs, and bias was reviewed for all included studies.

However, there are some limitations to our findings. First, although includable, there were no eligible randomized studies available for telehealth treatment of some less common mental health conditions, such as schizophrenia, bipolar disorders, and personality disorders. This limits the generalizability of these findings across these serious mental health concerns. To assess whether treatment of these conditions is feasible by telehealth, evidence beyond randomized trials should be examined or further high-quality research primary conducted. Second, we only included studies of therapies delivered verbally via telephone or video, as this is most similar to the face-to-face nature of primary care, and we intentionally excluded chat-based or self-guided internet therapy modalities. There is emerging evidence to support the efficacy of chat for mental health treatment services [51,52]. There is also a growing body of work on internet-based therapies for the treatment of psychological conditions such as addictions [53], eating disorders [54], and depression [55] and for the delivery of specific therapies such as CBT [56]. Therefore, although these therapeutic approaches are outside the scope of this review, the role of chat-based or self-guided internet therapy cannot be discounted for remote management of mental health difficulties. Third, most included trials were conducted in the United States, with 2 from the United Kingdom. These health care systems may not be comparable in other countries or regions [57], which limits the generalizability of our findings across medical systems internationally. Fourth, the risk of bias in included studies was largely unclear. We were unable to conduct prespecified subgroup analysis excluding studies at high risk of bias, owing to the small number of studies eligible for inclusion. The possibility of risk of bias in included studies should be considered when interpreting these results. Fifth, we included both telephone and video modalities as telehealth and did not conduct a sensitivity analysis to test any differences between these modalities owing to the small number of included studies. It is possible that there may be differences between telephone and video telehealth care, and future studies may explore this. Sixth, although we anticipated heterogeneity and used a random-effects model, some measures of heterogeneity are high. In each of these cases, the maximum number of studies available at the time point was 3, and it is thought that even when appropriate, meta-analysis with a small number of included studies can lead to fluctuations in the *I*^2^ statistic and should be interpreted with caution [58]. The small number of included studies precluded explorations into heterogeneity, so it is unclear whether heterogeneity observed is solely due to variation between included studies or whether instability of the *I*^2^ statistic due to the small number of included studies inflated the estimate. Regardless, the presence of heterogeneity highlights differences between included studies and reinforces the need for large, high-quality studies exploring psychotherapy delivered via telehealth versus face-to-face care for less common mental illnesses. Seventh, although the outcomes selected are appropriate for the study question and aims, they are all clinician or patient self-report measures, which are subject to measurement and other biases. Finally, the maximum follow-up time for included studies was 12 months, and there was variability in the follow-up periods among studies. The management of mental illness can be chronic or lifelong, so our results do not speak to the effectiveness of telehealth for longer-term management of these conditions.

### Clinical and Research Implications

There are some important clinical implications of this research. To date, there has been some reported hesitancy from clinicians to use telehealth in their practice [6]. This appears driven by care providers rather than care receivers; patients report equal satisfaction and experience of therapeutic alliance when receiving individual care via telehealth versus face-to-face [59]. Therapist hesitancy may be due to lack of training, concerns about the quality of the therapeutic alliance including rapport building, ethical concerns around risk management, and technological limitations [7,60-62]. Given the increasing body of evidence demonstrating the similarity of mental health care delivered via telehealth compared with face-to-face, it is critical that therapist barriers toward telehealth modalities be addressed. This may take various potential forms, including the provision of training for the delivery of specific therapies via telehealth, which could be incorporated into professional development or tertiary training. Furthermore, regulatory bodies (eg, Australian Health Practitioner Regulation Agency in Australia) could also provide support and advice for the implementation of telehealth infrastructure such as billing processes or technical logistics.

In addition to these clinical implications, there are several possible directions for further research. Given the diverse range of patient populations, therapies, and psychological conditions that may be treated using telehealth, and the multiple modes of care delivery (ie, telephone, video, or blended), it may be beneficial to investigate how to optimize telehealth therapy for various patient groups. Future considerations could include understanding whether certain conditions are better suited to video or telephone delivery and whether telehealth is as effective when treating complex or comorbid mental illnesses and identifying whether there are any groups for which telehealth is not recommended. Developing specific and structured protocols or guidelines for the delivery of psychotherapy via telehealth to diverse patient groups will help ensure the consistent provision of best-practice telehealth care.

### Conclusions

The COVID-19 pandemic has pushed telehealth to the forefront of mental health care out of necessity. This review shows that there is insufficient evidence of difference between psychotherapy delivered via telehealth and psychotherapy delivered via face-to-face care for the management of less common mental and physical health conditions requiring psychological support. There was insufficient evidence of difference between groups across patient, process, and cost outcomes, including symptom severity, improvement, function, therapeutic working alliance, satisfaction, and cost. However, CIs often included the minimally important difference, so we cannot conclude whether psychotherapy delivered via telehealth versus via face-to-face are equivalent. Further research is needed to assess the efficacy of telehealth for some conditions for which this review found no evidence (such as schizophrenia and bipolar disorders) and to optimize the delivery of telehealth interventions across diverse patient groups. The current evidence indicates that psychotherapy delivered via telehealth may be an alternative to face-to-face psychotherapy for the treatment of less common mental health conditions and physical conditions requiring psychological care.

## Appendix

Multimedia Appendix 1 Process and financial outcomes.

Multimedia Appendix 2 Search strings.

Multimedia Appendix 3 Table of excluded studies.

Multimedia Appendix 4 Potentially relevant in-progress clinical trials.

Multimedia Appendix 5 Table of included scales.

## Abbreviations

CBT: cognitive behavioral therapy
PRISMA: Preferred Reporting Items for Systematic Reviews and Meta-Analyses
PTSD: posttraumatic stress disorder
RCT: randomized controlled trial
SMD: standardized mean difference
