# Supplementary material for: Telehealth Versus Face-to-face Psychotherapy for Less Common Mental Health Conditions: Systematic Review and Meta-analysis of Randomized Controlled Trials
Source: JMIR Ment Health. 2022 Mar 11;9(3):e31780. doi: 10.2196/31780 (PMC8956990; doi:10.2196/31780)
Supplement: Multimedia Appendix 1 [file mental_v9i3e31780_app1.docx]

## Appendix 1: Process and financial outcomes

#### Working alliance / therapeutic quality

##### Working alliance – client

The client-based working alliance scores were evaluated using three different scales, see Multimedia appendix 4 for a summary of scales used.

Five studies reported sufficient data for this outcome; 3 (involving 223 participants) were able to be pooled (Figure 6). Data was analysed at one time-point; immediately post-treatment. There was no difference between telehealth and face-to-face therapy, standardised mean difference was 0.11 (95% CI -0.34 to 0.57, *P =.*63). This subgroup had moderate-high levels of heterogeneity (I^2^ = 63%).

***
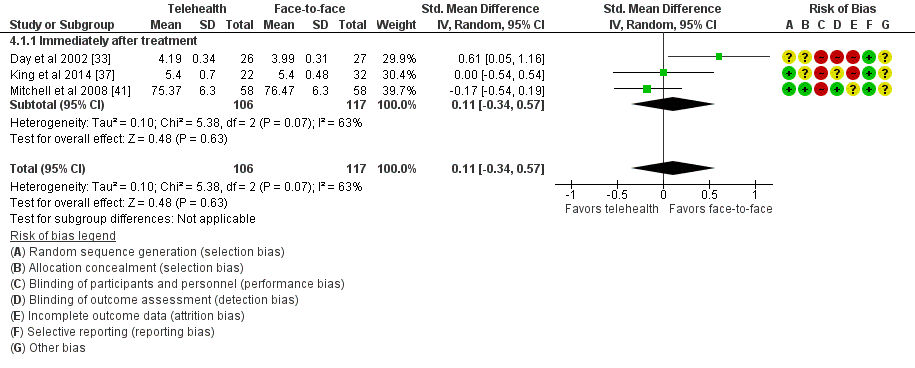
***

*Figure S1. Telehealth vs. Face-to-Face for mental conditions: assessment of client measures. Std: standard. [33, 37, 41]*

Freeman 2013 also reported youth and parent WAI scores, although standard deviation was not reported [35]. WAI was scored from 36 to 252, with higher scores denoting a better working alliance. A total of 32 youth in the telehealth group reported a mean WAI score of 215.9, while 39 participants in the face-to-face group had a mean score of 210.8. The mean WAI score of parents in the telehealth group was 224.8, and 221.6 in the face-to-face group. There was no difference between groups for both the youth and the parent scores. Xie 2013 [43] reported an approximation of working alliance by reporting a communication score on a Likert scale. Nine participants in the telehealth group immediately post-treatment rated communication at a mean of 11.5 (SD 1.4), while 13 participants in the face-to-face group reported a mean of 12 (SD 0.0). While the direction of the scale is unclear, there is no significant difference between groups in judgement of communication. There appears to be no difference between telehealth and face-to-face modalities for working alliance from the client/parent perspective.

##### Working alliance – therapist

The therapist-focused working alliance score was assessed using 2 scales, see Multimedia appendix 4 for a summary of scales used.

Two studies (104 participants in total) reported data on this outcome and were meta-analysed (Figure 7). Outcome was evaluated at a single time-point - immediately post-treatment. We did not find evidence of a difference between the two groups, mean difference was -0.16 (95% CI -0.91 to 0.59, *P =.*67). Heterogeneity was high (I^2^ = 72%).

***
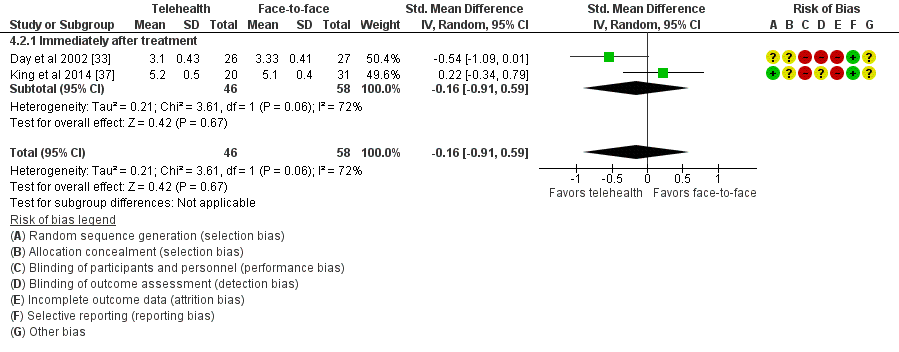
***

*Figure S2. Telehealth vs. Face-to-Face for mental conditions: assessment of therapist measures. Std: standard. [33, 37]*

#### Client satisfaction

The outcome was assessed using three different scales including: the CSS, CSQ, and the usefulness scale, see Multimedia appendix 4 for a summary of scales used.

Seven studies reported this outcome; three were able to be meta-analysed (Figure 8). These three studies involved a total of 131 participants. Data was assessed at one time-point - immediately post-treatment. Overall, we did not find evidence of a difference between the two groups, standardised mean difference was 0.12 (95% CI -0.3 to 0.53, *P =.*58).

***
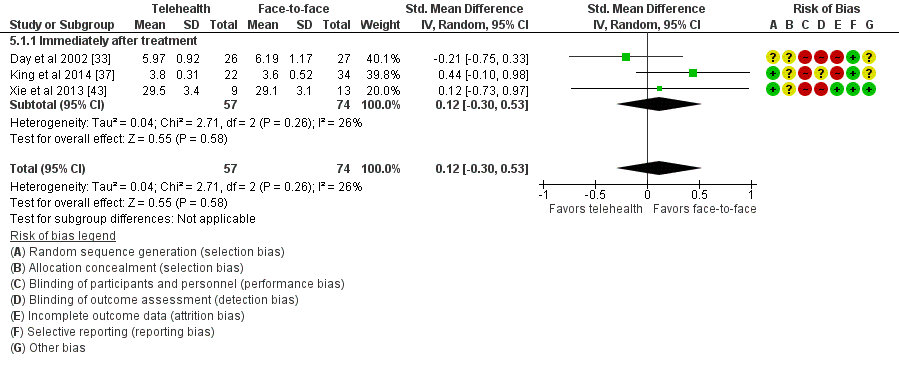
***

*Figure S3. Telehealth vs. Face-to-Face for mental conditions: assessment of satisfaction. Std: standard. [33, 37, 43]*

Four other studies also reported on some elements of client satisfaction. Burgess 2012, reported no significant difference between telehealth and face-to-face for client satisfaction with treatment, although raw values were not reported (*P =.*59 at post treatment; [31]). Comer 2017 reported satisfaction using the CSQ-8, finding no difference in client satisfaction scores between the groups for telehealth (18 participants, mean = 30.1) and face-to-face (17 participants, mean = 28.5). Both groups were highly satisfied with treatment; the highest score on CSQ-8 is 32 [32]. In addition to reporting client satisfaction scores included in the data synthesis, Day 2002 also reported therapist satisfaction scores using the TSS scale [33]. They found no significant difference in therapist satisfaction between telehealth (26 video participants, 5.7 (SD 0.89); 27 telephone participants, 5.4 (SD 1.3)) and face-to-face groups (27 participants 5.90 (SD 0.90). Watson 2017 [42] reported a Likert satisfaction measure; amongst telehealth participants, 28 out of 40 (70%) were very satisfied with treatment overall, and 36 out of 40 (90%) said the treatment had, at least somewhat, helped them. Similarly, face-to-face participants, 23 out of 33 (64%) were very satisfied with treatment overall and 31 out of 33 (94%) said they found that the treatment had, at least somewhat, helped them. Significance of the differences between groups was not reported. Client and therapist satisfaction with telehealth treatment appears equivalent to face-to-face satisfaction.

#### Cost

Three studies reported on the costs of telehealth versus face-to-face care. A multiple-choice procedure questionnaire (MCP) to assess monetary value to the patient found that those in the telehealth group rated the value per session higher than those in the face-to-face group immediately post-treatment (USD$17.50 telehealth vs USD$14.75 face-to-face; [37]). Mitchell 2008 [41], (analysis by Crow 2009 [39]), evaluated the costs of delivering treatment to patients. Treatment costs for the provider were lower in the telehealth group than in the face-to-face group for both cost per subject (USD$1648.48 telehealth vs USD$2684.38 face-to-face) and cost per abstinent subject (those for which treatment was effective; USD$7300.40 telehealth vs USD$9324 face-to-face [39, 41]). They concluded that telehealth is more cost effective than face-to-face treatment when conducted over large geographical areas. Watson 2017 also reported that the cost of therapists’ time was equivalent between the telehealth and face-to-face conditions, although no numerical data was reported [42].
