# Supplementary material for: Telehealth Versus Face-to-face Psychotherapy for Less Common Mental Health Conditions: Systematic Review and Meta-analysis of Randomized Controlled Trials
Source: JMIR Ment Health. 2022 Mar 11;9(3):e31780. doi: 10.2196/31780 (PMC8956990; doi:10.2196/31780)
Supplement: Multimedia Appendix 2 [file mental_v9i3e31780_app2.docx]

## Appendix 2: Search string

### Searches for RCTs

**PubMed Search run 18/11/2020**

("Telemedicine"[Mesh] OR "Videoconferencing"[Mesh] OR Telehealth[tiab] OR Telemedicine[tiab] OR Videoconferencing[tiab] OR ((Telephone[tiab]) AND (Consultation[tiab] OR face-to-face[tiab] OR in-person[tiab])) OR telephone-delivered[tiab])

AND

("Primary Health Care"[Mesh] OR "General Practice"[Mesh] OR rehabilitation[sh] OR "Outpatients"[Mesh] OR "Speech Therapy"[Mesh] OR Outpatient[tiab] OR “Primary health”[tiab] OR “Primary care”[tiab] OR “General practice”[tiab] OR “General practices”[tiab] OR “General practitioners”[tiab] OR “General practitioner”[tiab] OR “Family practice”[tiab] OR Physician[tiab] OR Physicians[tiab] OR Clinician[tiab] OR Clinicians[tiab] OR Therapist[tiab] OR Nurse[tiab] OR Nurses[tiab] OR Physiotherapist[tiab] OR Rehabilitation[tiab] OR Diabetes[tiab] OR Diabetic[tiab] OR Asthma[tiab] OR Depression[tiab] OR “Ïrritable bowel”[tiab] OR IBS[tiab] OR PTSD[tiab] OR “Chronic fatigue”[tiab])

AND

((Face-to-face[tiab]) OR “Usual care”[tiab] OR Visits[tiab] OR Visit[tiab] OR In-person[tiab] OR “In person”[tiab] OR ((Clinic[tiab] OR Centre[tiab] OR Home[tiab]) AND (Based[tiab] OR Contact[tiab])) OR Conventional[tiab] OR “Practice-based”[tiab] OR “Practice based”[tiab] OR Traditional[tiab] OR “Standard care”[tiab] OR Homecare[tiab] OR ((Routine[tiab] OR Home[tiab]) AND (Care[tiab])))

AND

("Delivery of Health Care"[Mesh] OR Delivery[tiab] OR Delivered[tiab] OR Via[tiab] OR Received[tiab])

AND

("Treatment Outcome"[Mesh] OR "Patient Satisfaction"[Mesh] OR Therapy[sh] OR Diagnosis[sh] OR “Clinical outcomes”[tiab] OR Treatment[tiab] OR Diagnostic[tiab] OR Efficacy[tiab])

AND

(Randomized controlled trial[pt] OR controlled clinical trial[pt] OR randomized[tiab] OR randomised[tiab] OR placebo[tiab] OR "drug therapy"[sh] OR randomly[tiab] OR trial[tiab] OR groups[tiab])
NOT

(Animals[Mesh] not (Animals[Mesh] and Humans[Mesh]))

NOT

(“Case Reports”[pt] OR Editorial[pt] OR Letter[pt] OR Meta-Analysis[pt] OR “Observational Study”[pt] OR “Systematic Review”[pt] OR “Case Report”[ti] OR “Case series”[ti] OR Meta-Analysis[ti] OR “Meta Analysis”[ti] OR “Systematic Review”[ti] OR “Systematic Literature Review”[ti] OR “Qualitative study”[ti] OR Protocol[ti])

**CENTRAL via the Cochrane Library run 18/11/2020**

([mh Telemedicine] OR [mh Videoconferencing] OR Telehealth:ti,ab OR Telemedicine:ti,ab OR Videoconferencing:ti,ab OR ((Telephone:ti,ab) AND (Consultation:ti,ab OR “ face-to-face”:ti,ab OR “in person”:ti,ab)) OR “telephone delivered”:ti,ab)

AND

([mh "Primary Health Care"] OR [mh "General Practice"] OR [mh Outpatients] OR [mh "Speech Therapy"] OR Outpatient:ti,ab OR "Primary health":ti,ab OR "Primary care":ti,ab OR "General practice":ti,ab OR "General practices":ti,ab OR "General practitioners":ti,ab OR "General practitioner":ti,ab OR "Family practice":ti,ab OR Physician:ti,ab OR Physicians:ti,ab OR Clinician:ti,ab OR Clinicians:ti,ab OR Therapist:ti,ab OR Nurse:ti,ab OR Nurses:ti,ab OR Physiotherapist:ti,ab OR Rehabilitation:ti,ab OR Diabetes:ti,ab OR Diabetic:ti,ab OR Asthma:ti,ab OR Depression:ti,ab OR "Ïrritable bowel":ti,ab OR IBS:ti,ab OR PTSD:ti,ab OR "Chronic fatigue":ti,ab)

AND

(("Face-to-face":ti,ab) OR "Usual care":ti,ab OR Visits:ti,ab OR Visit:ti,ab OR "In person":ti,ab OR ((Clinic:ti,ab OR Centre:ti,ab OR Home:ti,ab) AND (Based:ti,ab OR Contact:ti,ab)) OR Conventional:ti,ab OR "Practice based":ti,ab OR Traditional:ti,ab OR "Standard care":ti,ab OR Homecare:ti,ab OR ((Routine:ti,ab OR Home:ti,ab) AND (Care:ti,ab)))

AND

([mh "Delivery of Health Care"] OR Delivery:ti,ab OR Delivered:ti,ab OR Via:ti,ab OR Received:ti,ab)

AND

([mh "Treatment Outcome"] OR [mh "Patient Satisfaction"] OR "Clinical outcomes":ti,ab OR Treatment:ti,ab OR Diagnostic:ti,ab OR Efficacy:ti,ab)

**Embase search run 18/11/2020**

('Telemedicine'/exp OR 'Videoconferencing'/exp OR Telehealth:ti,ab OR Telemedicine:ti,ab OR Videoconferencing:ti,ab OR ((Telephone:ti,ab) AND (Consultation:ti,ab OR face-to-face:ti,ab OR in-person:ti,ab)) OR telephone-delivered:ti,ab)

AND

('Primary Health Care'/exp OR 'General Practice'/exp OR 'Outpatient'/exp OR 'Speech Therapy'/exp OR Outpatient:ti,ab OR "Primary health":ti,ab OR "Primary care":ti,ab OR "General practice":ti,ab OR "General practices":ti,ab OR "General practitioners":ti,ab OR "General practitioner":ti,ab OR "Family practice":ti,ab OR Physician:ti,ab OR Physicians:ti,ab OR Clinician:ti,ab OR Clinicians:ti,ab OR Therapist:ti,ab OR Nurse:ti,ab OR Nurses:ti,ab OR Physiotherapist:ti,ab OR Rehabilitation:ti,ab OR Diabetes:ti,ab OR Diabetic:ti,ab OR Asthma:ti,ab OR Depression:ti,ab OR "Ïrritable bowel":ti,ab OR IBS:ti,ab OR PTSD:ti,ab OR "Chronic fatigue":ti,ab)

AND

(("Face-to-face":ti,ab) OR "Usual care":ti,ab OR Visits:ti,ab OR Visit:ti,ab OR In-person:ti,ab OR "In person":ti,ab OR ((Clinic:ti,ab OR Centre:ti,ab OR Home:ti,ab) AND (Based:ti,ab OR Contact:ti,ab)) OR Conventional:ti,ab OR Practice-based:ti,ab OR "Practice based":ti,ab OR Traditional:ti,ab OR "Standard care":ti,ab OR Homecare:ti,ab OR ((Routine:ti,ab OR Home:ti,ab) AND (Care:ti,ab)))

AND

('health care delivery'/exp OR Delivery:ti,ab OR Delivered:ti,ab OR Via:ti,ab OR Received:ti,ab)

AND

('Treatment Outcome'/exp OR 'Patient Satisfaction'/exp OR "Clinical outcomes":ti,ab OR Treatment:ti,ab OR Diagnostic:ti,ab OR Efficacy:ti,ab)

AND

(random* OR factorial OR crossover OR placebo OR blind OR blinded OR assign OR assigned OR allocate OR allocated OR 'crossover procedure'/exp OR 'double-blind procedure'/exp OR 'randomized controlled trial'/exp OR 'single-blind procedure'/exp NOT ('animal'/exp NOT ('animal'/exp AND 'human'/exp)))

 AND [embase]/lim

**Clinicaltrials.gov**

Intervention field: (Telemedicine OR Videoconferencing OR Telephone OR Telehealth) AND (“Usual care” OR “Standard care” OR Face-to-face OR Face-to-face”)

Condition or disease field: “Behavior disorder” OR “defiant disorder” OR “Bipolar disorder” OR “Dissociative disorders” OR “Opioid dependence” OR “Drug dependence” OR “Substance abuse” OR “Alcohol abuse” OR Bulimia OR eating disorder OR Anorexia OR “Binge eating”

OR

Intervention field: (Telemedicine OR Videoconferencing OR Telephone OR Telehealth) AND (“Usual care” OR “Standard care” OR Face-to-face OR Face-to-face”)

Condition or disease field: ADHD OR “Psychological support” OR “Psychological therapies” OR “Psychological therapy” OR Autism OR Schizophrenia OR Psychosis OR “Personality disorders”

**WHO ICTRP**

Telemedicine AND Disorder OR Telehealth AND Disorder OR Videoconferencing AND Disorder

Telemedicine AND Substance abuse OR Telehealth AND Substance abuse  OR Videoconferencing AND Substance abuse

Telemedicine AND Bulimia OR Telehealth AND Bulimia  OR Videoconferencing AND Bulimia

Telemedicine AND  Anorexia OR Telehealth AND Anorexia  OR Videoconferencing AND Anorexia

Telemedicine AND  ADHD OR Telehealth AND ADHD  OR Videoconferencing AND ADHD

Telemedicine AND  Psychological  OR Telehealth AND Psychological   OR Videoconferencing AND Psychological

Telemedicine AND  Autism  OR Telehealth AND Autism   OR Videoconferencing AND Autism
