# Supplementary material for: Telehealth Versus Face-to-face Psychotherapy for Less Common Mental Health Conditions: Systematic Review and Meta-analysis of Randomized Controlled Trials
Source: JMIR Ment Health. 2022 Mar 11;9(3):e31780. doi: 10.2196/31780 (PMC8956990; doi:10.2196/31780)
Supplement: Multimedia Appendix 3 [file mental_v9i3e31780_app3.docx]

## Appendix 3: Table of Excluded Studies

| **Reference** | | **Reason for exclusion** |
| --- | --- | --- |
| *Forward and Backward Citation Analysis* | | |
| 1 | Marrone S, Mitchell JE, Crosby R, Wonderlich S, Jollie-Trottier T. Predictors of response to cognitive behavioral treatment for bulimia nervosa delivered via telemedicine versus face-to-face. Int J Eating Disord. 2009;42(3):222-7. | Outcomes |
| 2 | McKay JR, Van Horn DHA, Oslin DW, Lynch KG, Ivey M, Ward K, et al. A randomized trial of extended telephone-based continuing care for alcohol dependence: Within-Treatment substance use outcomes. J Consult Clin Psychol. 2010;78(6):912-23. | Comparator - groups not comparable |
| 3 | Mohr DC, Hart SL, Julian L, Catledge C, Honos-Webb L, Vella L, et al. Telephone-administered psychotherapy for depression. Arch Gen Psychiatry. 2005;62(9):1007-14. | Comparator - No face-to-face |
| 4 | Bambauer KZ, Aupont O, Stone PH, Locke SE, Mullan MG, Colagiovanni J, et al. The effect of a telephone counseling intervention on self-rated health of cardiac patients. Psychosomatic Medicine. 2005;67(4):539-45. | Comparator – groups not comparable |
| 5 | Cluver JS, Schuyler D, Frueh BC, Brescia F, Arana GW. Remote psychotherapy for terminally ill cancer patients. Journal of Telemedicine and Telecare. 2005;11(3):157-9. | Study design – not RCT |
| 6 | King SC, Richner KA, Tuliao AP, Kennedy JL, McChargue DE. A comparison between telehealth and face-to-face delivery of a brief alcohol intervention for college students. Substance Abuse. 2020;41(4):501-9. | Setting – not primary care |
| 7 | Watson HJ, McLagan N, Zerwas SC, Crosby RD, Levine MD, Runfola CD, et al. Cost-effectiveness of internet-based cognitive-behavioral treatment for bulimia nervosa: Results of a randomized controlled trial. J Clin Psychiatry. 2018;79(1). | Intervention – chat based |
| 8 | Glueckauf RL, Fritz SP, Ecklund-Johnson EP, Liss HJ, Dages P, Carney P. Videoconferencing-based family counseling for rural teenagers with epilepsy: Phase 1 findings. Rehabil Psychol. 2002;47(1):49-72. | Study design – not RCT |
| *Initial search* | | |
| 9 | Arundine A, Bradbury CL, Dupuis K, Dawson DR, Ruttan LA, Green REA. Cognitive behavior therapy after acquired brain injury: Maintenance of therapeutic benefits at 6 months posttreatment. Journal of Head Trauma Rehabilitation. 2012;27(2):104-12. | Study design – not RCT |
| 10 | Blasco S, Chirivella J, Chirivella C, Gagliardo P, Taylor P, Penadés V. Comparison between traditional neuropsychology treatment and the combination of traditional treatment with virtual reality. Brain Injury. 2016;30(5-6):686. | Study design – no full text |
| 11 | Blasco S, Chirivella J, Gagliardo P, Ferreiro T, Izquierdo R, Penadés V, et al. Comparison between traditional physiotherapy treatment and the combination of traditional treatment with virtual reality. Brain Injury. 2016;30(5-6):685. | Duplicate |
| 12 | Choi KH, Lee E, Seo HJ. Community-based multi-site randomized controlled trial of behavioral activation for patients with negative symptoms. Schizophrenia Bulletin. 2019;45:S140. | Study design – abstract only |
| 13 | Conwell Y, Simning A, Driffill N, Xia Y, Tu X, Messing SP, et al. Validation of telephone-based behavioral assessments in aging services clients. Int Psychogeriatr. 2018;30(1):95-102. | Study design – validation study |
| 14 | Farabee D, Calhoun S, Veliz R. An Experimental Comparison of Telepsychiatry and Conventional Psychiatry for Parolees. Psychiatr Serv. 2016;67(5):562-5. | Provider – psychiatrist |
| 15 | Freeman KA, Harris MA, Duke D, Boston B, Hirschfield B. Hey mikey, he likes it: Patient experiences with skype-based intervention for teens with poorly controlled diabetes. Diabetes. 2012;61:A205. | Duplicate |
| 16 | Hall DL, Lattie EG, Milrad SF, Czaja S, Fletcher MA, Klimas N, et al. Telephone-administered versus live group cognitive behavioral stress management for adults with CFS. J Psychosom Res. 2017;93:41-7. | Study design – not RCT |
| 17 | Kiropoulos LA, Klein B, Austin DW, Gilson K, Pier C, Mitchell J, et al. Is internet-based CBT for panic disorder and agoraphobia as effective as face-to-face CBT? J Anxiety Disord. 2008;22(8):1273-84. | Intervention - email |
| 18 | Lleras M, Casellas-Grau A, Sumalla E, Ortega AR, Andrés JMB, Ochoa C. Randomized Control Trial (RCT) of Online vs Presential Positive Group Psychotherpay. Psycho-Oncology. 2017;26:44-5. | Study design – abstract only |
| 19 | Malakouti SK, Nojomi M, Mirabzadeh A, Mottaghipour Y, Zahiroddin A, Kangrani HM. A Comparative study of nurses as case manager and telephone follow-up on clinical outcomes of patients with severe mental illness. Iranian Journal of Medical Sciences. 2016;41(1):19-27. | Comparator – groups not comparable |
| 20 | Man DW, Soong WY, Tam SF, Hui-Chan CW. Self-efficacy outcomes of people with brain injury in cognitive skill training using different types of trainer-trainee interaction. Brain Inj. 2006;20(9):959-70. | Intervention - online |
| 21 | Mathiasen K, Andersen TE, Riper H, Kleiboer AA, Roessler KK. Blended CBT versus face-to-face CBT: a randomised non-inferiority trial. BMC Psychiatry. 2016;16(1):432. | Study design - protocol |
| 22 | Mitchell J, Wonderlich S, Lancaster K. Randomized trial comparing telemedicine delivered cognitive behavioral therapy to in-person cognitive behavioral therapy in the outpatient treatment of bulimia nervosa. World psychiatric association, international congress 2006; july 12 - 16 2006; istanbul, turkey. 2006:107. | Study design – abstract only |
| 23 | Shepard DS, Daley MC, Neuman MJ, Blaakman AP, McKay JR. Telephone-based continuing care counseling in substance abuse treatment: Economic analysis of a randomized trial. Drug and Alcohol Dependence. 2016;159:109-16. | Comparator – groups not comparable |
| 24 | Shulman M, John M, Kane JM. Home-Based Outpatient Telepsychiatry to Improve Adherence With Treatment Appointments: A Pilot Study. Psychiatr Serv. 2017;68(7):743-6. | Provider - psychiatrist |
| 25 | Singh H, Sendejas M, Pallivathucal LB, Gonzalez M, Decker S, Pimentel AR, et al. Using telehealth to enhance engagement and reduce patient burden in the management of gestational diabetes: A randomized trial in a diverse community setting. Diabetes. 2019;68. | Study design – abstract only |
| 26 | Tse YJ, McCarty CA, Stoep AV, Myers KM. Teletherapy delivery of caregiver behavior training for children with attention-deficit hyperactivity disorder. Telemed J E Health. 2015;21(6):451-8. | Study design – not RCT |
| *Clinical Trial Registries* | | |
| 27 | Harder VS, Musau AM, Musyimi CW, Ndetei DM, Mutiso VN. A randomized clinical trial of mobile phone motivational interviewing for alcohol use problems in Kenya. Addiction. 2020 Jun;115(6):1050-60. | Provider – variable between participants |
| 28 | Fann JR, Bombardier CH, Vannoy S, Dyer J, Ludman E, Dikmen S, Marshall K, Barber J, Temkin N. Telephone and in-person cognitive behavioral therapy for major depression after traumatic brain injury: a randomized controlled trial. J Neurotrauma. 2015 Jan 1;32(1):45-57. | Study design – not true randomisation |
| 29 | Marino F, Chilà P, Failla C, Crimi I, Minutoli R, Puglisi A, Arnao AA, Tartarisco G, Ruta L, Vagni D, Pioggia G. Tele-Assisted Behavioral Intervention for Families with Children with Autism Spectrum Disorders: A Randomized Control Trial. Brain Sci. 2020 Sep 18;10(9):649. | Intervention |
