# Supplementary material for: Telehealth Versus Face-to-face Psychotherapy for Less Common Mental Health Conditions: Systematic Review and Meta-analysis of Randomized Controlled Trials
Source: JMIR Ment Health. 2022 Mar 11;9(3):e31780. doi: 10.2196/31780 (PMC8956990; doi:10.2196/31780)
Supplement: Multimedia Appendix 4 [file mental_v9i3e31780_app4.docx]

## Appendix 4: Potentially Relevant ‘in progress’ clinical trials

|  | *Clinical Trial Title* | *Trial Number* | *Status* |
| --- | --- | --- | --- |
| 1 | OT Intervention Via Telehealth for Children With ASD and ADHD | NCT03957993 | Completed 2019, no results posted |
| 2 | TIA: a feasibility study of telemedicine in addictions | ISRCTN36756455 | Completed 2020, no results posted |
