# Supplementary material for: Telehealth Versus Face-to-face Psychotherapy for Less Common Mental Health Conditions: Systematic Review and Meta-analysis of Randomized Controlled Trials
Source: JMIR Ment Health. 2022 Mar 11;9(3):e31780. doi: 10.2196/31780 (PMC8956990; doi:10.2196/31780)
Supplement: Multimedia Appendix 5 [file mental_v9i3e31780_app5.docx]

## Appendix 5: Table of Included Scales

| **Outcome** | **Abbreviated name** | **Full Name** | **Study** | **Purpose of scale** | **Quality** | **Items** | **Delivery** | **Scoring** |
| --- | --- | --- | --- | --- | --- | --- | --- | --- |
| **Severity of symptoms** | CFS | Chalder fatigue scale | Burgess | Measure of severity of fatigue in fatiguing illnesses | Valid and reliable | 11 | Self-reported measure | 4-point scale (less than usual; much more than usual), higher score indicates a higher severity of fatigue |
|  | CGI-S | Clinical Global Impression - Severity | Comer, Xie | General measure assessing treatment-related changes in functioning. | Valid and reliable | 1 | Clinician-rated measure | 7-point scale (1 normal; 7 among the most severely ill patients), higher scores indicate more severe symptoms |
|  | BSI | Brief symptom inventory | Day | Symptom checklist for psychological clients | Valid and reliable | 53 | Self-reported measure | 5-point scale (0 not at all; 4 extremely). Higher scores indicate higher symptom burden. |
|  | YGTSS | Yale Global Tic Severity Scale | Himle | Measure used to assess the severity of tic disorders | Valid and reliable | 5 sections | Clinician-rated measure | 5-point scale, (0 no symptoms - 50 high symptom burden), with higher numbers indicating more severe tics. |
|  | PHQ-15 | Patient Health Questionnaire-15 | McAndrew | Diagnostic measure to identify the number and types of symptoms a patient is experiencing | Valid and reliable | 15 | Self-reported measure | 3-point scale (0 not bothered; 2 bothered a lot), higher scores indicate a greater symptom burden (5 - low burden, 10 - medium burden, 15 - high burden) |
|  | HADS | Hospital Anxiety and Depression Scale | Watson | General measure used to detect anxiety and depressive disorders. | Valid and reliable | 14 | Self-reported measure | 3-point scale (0 - 3), higher scores indicate higher levels of anxiety and depression symptoms. |
| **Improvement** | CGI-I | Clinical Global Impression - Improvement | Xie, Comer, Himle | General measure assessing treatment response | Valid and reliable | 1 | Clinician-rated measure | 7-point scale (1 very much improved; 7 very much worse), higher scores indicate worse response to treatment |
|  | SR GIS | Self-rated Global Improvement | Burgess | Measure of self-assessed symptom severity | Custom - unvalidated | 6 | Self-reported measure | 6-point scale (“very much better”; “very much worse”), higher scores indicate worse levels of improvement or response to therapy |
|  | MAC - H/H | Mental Adjustment to Cancer: helpless/hopeless subscale | Watson | Measure of coping responses/attitudes of cancer patients | Valid and reliable | 40 | Self-reported measure | 4-point scale (1 definitely does not apply to me; 4 definitely applies to me), higher score indicates a stronger use of the coping strategy |
| **Function** | CGAS | Children's Global Assessment Scale | Comer, Xie | General measure of functioning in children | Valid and reliable | 1 | Clinician-rated measure | Scores range from 0-100, lower scores indicate greater impairment. |
|  | MOS | Medical outcomes survey | Burgess | General measure assessing extent of the impact of ill-health on normal activities | Valid and reliable | 6 | Self-reported measure | 3-point scale (1 limited for more than 6 months; 3 not limited at all). A higher score indicates less physical limitation. |
|  | GAF | Global assessment of functioning | Day | Measure of function in patients with mental illness | Valid and reliable | 10 sections | Clinician-rated measure | Score of 1 - 100, 100 being the highest functioning. |
|  | VR-36 | Veterans RAND | McAndrew | Measure of health-related quality of life used in medically ill veterans | Valid and reliable | 36 | Self-reported measure | 5-point scale (no, none of the time - yes, all of the time). Scores of 0 - 100, higher scores indicate higher levels of function. |
|  | SF-36 | 36-item Short Form Survey | Mitchell | General measure of health-related quality of life. | Valid and reliable | 36 | Self-reported measure | Yes/No scale. Scores of 0 - 100, higher scores indicate higher levels of function. |
| **Working alliance** | VPPS | Vanderbilt psychotherapy process scale – participation sub-total | Day | General scale used to assess aspects of patient and therapist behaviours and attitudes during therapy. | Valid and reliable | 80 | Both patient and clinician rated | Scores are transformed - I think higher scores are better. |
|  | HAQ-II - client and therapist | Helping alliance questionnaire | King 2014 | General measure of the quality of the therapeutic alliance | Valid and reliable | 19 | Both patient and clinician rated | 6-point Likert scale (1 - 6), a higher score indicates higher satisfaction |
|  | WAI - client | Working Alliance Inventory - Client score | Mitchell, Freeman | General measure of the strength of the collaborative relationship between the client and the clinician | Valid and reliable | 36 | Self-reported measure | 7-point scale, (1 never; 7 always), scoring range of 36–252. Higher scores indicate a more positive therapeutic alliance. |
| **Satisfaction** | CSS | Client satisfaction scale | Day | General scale used to assess satisfaction with the therapeutic experience. | Valid and reliable | 7 | Self-report | The higher the score, the higher satisfaction |
|  | CSQ-8 | Client satisfaction questionnaire | King 2014, Comer | General measure used to assess overall satisfaction with treatment | Valid and reliable | 8 | Self-reported measure | 4-point scale (1 - 4), a higher score indicates higher satisfaction. |
|  | Usefulness | Self-designed Likert scale measuring usefulness | Xie | Measure of the degree of acceptance of the intervention | Custom - unvalidated | 1 | Self-reported measure | 4-point scale (less than usual; much more than usual), higher score indicates a higher severity of fatigue |
|  | TAQ | Treatment acceptability questionnaire | Himle | Measure of the acceptability of psychological treatments for adults and children | Valid and reliable | 6 | Self-reported measure | Scale range 7 - 42, higher scores indicate higher levels of treatment acceptability. |
|  | TSS | Therapist satisfaction scale | Day | General scale used to assess satisfaction with the therapeutic experience. | Valid and reliable | 7 | Self-report | The higher the score, the higher satisfaction |
